# Editorial: Innovation in surgery and surgical education

**DOI:** 10.3389/fsurg.2024.1461067

**Published:** 2024-07-24

**Authors:** Marina Yiasemidou

**Affiliations:** Department of Colorectal Surgery, The Royal London Hospital, Barts Health NHS Trust, London, United Kingdom

**Keywords:** innovation, education, training, surgery, sustainability, collaboration, robotic surgery, laparoscopic surgery

**Editorial on the Research Topic** Innovation in surgery and surgical education

The face of surgery is changing at a phenomenally rapid rate. Shifts in societal perceptions, coupled with a better understanding of the benefits of global collaboration and the use of educational technology, are driving positive changes in our field.

In the current topic, the concept of clinical innovation is being explored. Binyu et al. described their novel method for closing the inner ring of a Gilbert type III indirect inguinal hernia. In this randomized controlled trial, patients were allocated to closed and non-closed groups. They concluded that closure reduces the incidence of postoperative seroma and postoperative pain without increasing the risk of postoperative infection and recurrence. Furthermore, Zou et al. created a validated questionnaire aiming to address the very important issue of prevention of intraoperative-acquired pressure injuries, a valuable tool for increasing intraoperative patient safety. Iyad et al. described three five millimeter ports, which reduced the number of reusable instruments and ports. The novel technique had equivalent in-patient outcomes to traditional laparoscopic cholecystectomy but was more cost effective. Although not highlighted by the authors, the technique may be more sustainable than the conventional one.

Sustainability was the topic in two of the original papers published in this issue, with Westwood et al. informing us about the application of the Royal College of Surgeons Green Theatre Checklist. Lathan et al. assessed the impact of telemedicine, a groundbreaking intervention popularized during the COVID-19 pandemic, on the reduction of carbon footprint. Telemedicine was found to be sustainable and safe in the diagnosis of post-operative surgical site infection (SSI). Moreover, the prevention of SSI was the topic of a future global collaborative study, announced by Heinz et al. in their protocol for a pan-specialty survey of SSI prevention practices.

Surgical education also featured in this issue, with Georgiou et al. using biomarkers to demonstrate the impact of simulation on reducing trainee stress levels. The reader also had the opportunity to find out about the progression, current status, and future of surgical training in the Caribbean Newnham et al.

The issue was complimented by two narrative reviews, one of which was by Walshaw et al. describing the evolution of minimally invasive surgery, including a critical view of relevant landmark studies assessing its efficiency. Finally, Dexter et al. provided a comprehensive review of the pathophysiology, diagnosis, and treatment of fecal incontinence, describing traditional and cutting-edge treatments.

In summary, the current issue is inclusive of research from parts of the globe that at times are not proportionally represented in publications. It touched upon topical and very important issues, such as global collaboration and sustainability, whilst providing a condensed wealth of knowledge in other topics through comprehensive reviews. The diversity of the topics within this issue demonstrates that innovation can be achieved in every aspect of clinical and educational practice.

